# Gardenoside Hinders Caspase-1-Mediated Hepatocyte Pyroptosis Through the CTCF/DPP4 Signaling Pathway

**DOI:** 10.3389/fphys.2021.669202

**Published:** 2021-09-08

**Authors:** Tian Shen, Tao Lei, Lin Chen, Bing-Bing Zhu, Bi-Lin Xu, Cui-Ping Zhang, Hong-Ping Wang

**Affiliations:** ^1^Department of Endocrinology, Putuo Hospital, Shanghai University of Traditional Chinese Medicine, Shanghai, China; ^2^Department of Nephrology, Putuo Hospital, Shanghai University of Traditional Chinese Medicine, Shanghai, China

**Keywords:** gardenoside, NAFLD, inflammasome, caspase-1, pyroptosis, CTCF

## Abstract

Non-alcoholic fatty liver disease (NAFLD)is accompanied by typical inflammatory damage and cell death. As a pro-inflammatory form of cell death, pyroptosis participates in important pathological processes involved in NAFLD. Regulatory roles of both CCCTC-binding factor (CTCF) and dipeptidyl peptidase-4 (DPP4) have been reported in NAFLD, but it is still unclear whether the mechanism of action of gardenoside, a potential therapeutic for NAFLD, can be driven *via* these proteins. In this study, the direct interaction between CTCF and DPP4 was first confirmed by a dual-luciferase reporter assay system. Then, a cell model of NAFLD was established by induction with palmitic acid (PA) and lipopolysaccharide (LPS). A mouse NAFLD model was established, and the effect of gardenoside on both the cell and mouse models of NAFLD was also investigated. Increased lipid accumulation, NLRP3 inflammasome activation, and hepatocyte pyroptosis were recorded in NAFLD *in vitro* and *in vivo*. Gardenoside treatment effectively reduced the lipid accumulation, increased cell viability, reduced reactive oxygen species (ROS) generation, and attenuated pyroptosis and apoptosis in NAFLD in the *in vitro* and *in vivo* models. Alterations in these biological processes were evidenced by the decreased expression levels of several pro-pyroptotic markers including the NLR family, pyrin domain-containing 3 (NLRP3), apoptosis-related speckle-like protein (ASC), caspase-1 p20, Gasdermin D N-terminal domain (GSDMD-N), and IL-1β, along with simultaneously decreased CTCF and DPP4 levels. Importantly, CTCF silencing or DPP4 silencing exhibited effects similar to gardenoside treatment, while CTCF overexpression counteracted this trend, which indicated that CTCF might be a target responsible for gardenoside-induced alleviation of NAFLD, such therapeutic effects might be achieved through controlling the expression of the direct target of CTCF (DPP4) and several downstream molecules. In general, the current study provides a promising strategy for NAFLD treatment.

## Introduction

Non-alcoholic fatty liver disease (NAFLD) is a clinicopathologic syndrome characterized by steatosis of liver parenchyma and excessive accumulation of lipid in hepatocytes. The pathogenesis of the NAFLD is closely associated with obesity, hyperlipidemia, and insulin resistance (Anavi et al., 2017; Zheng et al., 2018; Sun et al., 2020; Wang et al., 2020). The treatment of NAFLD is challenging in clinical settings due to the lack of knowledge on the complete mechanism underlying its pathogenesis. Thus, elucidating the molecular pathogenesis of NAFLD and finding alternative drugs targeting the pathways involved are of critical importance.

Previous works demonstrated that NAFLD is accompanied by typical inflammatory injury and cell death (Arrese et al., 2016). As a form of pro-inflammatory cell death, pyroptosis participates in the damage process involved in NAFLD (Beier and Banales, 2018). Several studies reported that pyroptosis in NAFLD is mainly mediated by the caspase-1 classical pyroptosis pathway (Qiu et al., 2018; Wu et al., 2019). In this pathway, apoptosis-related speckle-like protein (ASC), absent in melanoma 2 (AIM2), NLR family pyrin domain containing 3 (NLRP3), and other pattern recognition receptors (PRRs) combine with pro-caspase-1 to form high-molecular inflammasome. Then, the inflammasome activates caspase-1, stimulates the maturation of IL-18 precursor and IL-1β precursor, and cleaves the downstream protein gasdermin-D (GSDMD) to release the active N-terminal disability fragment (GSDMD-N), which leads to the occurrence of cell pyroptosis (Barker et al., 2011; Compan et al., 2015). Recent research results have shown that the increased expression of NLRP3 inflammasome in NAFLD was closely related to the pathogenesis of NAFLD (Wan et al., 2016; Thomas, 2017). It was reported that hyperglycemia and reactive oxygen species (ROS) caused by diabetes result in abnormal activation of NLRP3 inflammasome (Mathew, 2018; Oguntibeju, 2019), which, in turn, promotes liver fibrosis, cirrhosis, liver cell apoptosis, and pyroptosis.

Dipeptidyl peptidase-4 (DPP4) is an aminopeptidase (Klemann et al., 2016) that is widely distributed in the body; the soluble form of DPP4 (sDPP4) in the plasma exerts complex biological effects (Wronkowitz et al., 2014). DPP4 regulates the activity of a variety of biological peptides and participates in a variety of pathological and physiological processes, such as immune stimulation and extracellular matrix connection (Röhrborn et al., 2015). For example, Miyazaki et al. (2012) studied liver tissues of 17 patients with NAFLD and found that the DPP4 gene expression level in normal liver tissues was significantly lower than that of the liver tissues of patients. Several studies have found that serum DPP4 levels in patients with NAFLD were related to liver injury markers γ-Glutamyl transpeptidase (GGT), glutamine synthetase (GLUL), and liver histological changes (Tsai et al., 2017; Barchetta et al., 2020), suggesting that liver fat formation and liver damage may be related to the expression of DPP4 in liver tissue. DPP4 inhibitors play a vital role in the regulation of obesity and NAFLD. Iwasaki et al. (2012) found a significant improvement of hemoglobin A1c (HbAlc), alanine aminotransferase, aspartate aminotransferase, and γ-gluten Aminoacyl transpeptidase in 30 patients with both fatty liver and type 2 diabetes mellitus (T2DM) after treatment by DPP4 inhibitor sitagliptin for 4 months. In addition, Birnbaum et al. (2019) demonstrated that compared with the control group, the expression levels of ASC, NLRP3, IL-1β, and IL-6 were lower in diabetic mice after linagliptin intervention, indicating that the number of NLRP3/ASC inflammasome in diabetic mice may be related to the expression level of DPP4. However, it is unclear whether DPP4 is involved in NAFLD-inflammasome.

CCCTC-binding factor (CTCF) is a gene known for its chromatin-organizing and transcription factor properties. A previous study in liver diseases showed that CTCF combines with maternal hypomethylation imprinting control region (ICR) to promote the expression of lncRNA H19, which is a regulator of fatty liver, fibrosis, and other liver diseases (Li and Liu, 2020), suggesting that CTCF may play a role in NAFLD. Results from previous studies suggest a probable connection between DPP4 and CTCF (Zuin et al., 2014; Li et al., 2018). However, the regulation mechanism of CTCF in NAFLD and its correlation with DPP4 remains unclear.

Gardenoside is the most effective of the bioactive molecules of the medicinal plant *Gardenia jasminoides* Ellis which has long been used in Chinese medicine for its hepatoprotective, antipyretic, and analgesic properties (Miura et al., 1996; Wang et al., 2015; Zhang et al., 2017). Previous studies have shown that gardenoside plays an important role in obesity and NAFLD. It has been shown to be effective for the treatment of NASH and inhibits free fatty acid-induced steatosis in hepatocytes by inhibiting inflammatory cytokines (Liang et al., 2015). However, the mechanism of gardenoside in NAFLD-associated inflammasome is not well elucidated. In addition, since the CTCF/DPP4 axis may be a potential inflammasome regulatory pathway, it is hypothesized that the mechanism of action of gardenoside may be mediated by its effect on pyroptosis through the CTCF/DPP4 pathway, which needs an in-depth verification.

Thus, in the present study, to explore the therapeutic effect of gardenoside in NAFLD and the molecular mechanism involved, we used *in vitro* and *in vivo* NAFLD models to validate the interaction between CTCF and DPP4 and evaluated the relevant biological processes, such as lipid accumulation, intracellular ROS production, NLRP3 inflammasome, and hepatocyte pyroptosis. We also explored the possible regulation of the CTCF/DPP4-NLRP3 regulatory network by gardenoside in NAFLD.

## Materials and Methods

### Bioinformatics Analysis

DNA binding motifs of DPP4 were discovered by the bioinformatics tool MEME suite (Bailey et al., 2015), and the DNA-binding motifs of CTCF interacting with DPP4 in a sequence-specific manner were predicted by the open-access database JASPAR (http://jaspar.genereg.net) (Fornes et al., 2020).

### Dual-Luciferase Reporter Assay

The dual-luciferase reporter assay was performed to verify the *in silico* prediction results. The wild-type (WT) and mutant (MUT) promoter regions of DPP4 were amplified and cloned into pGL3-basic luciferase vector to construct pGL3-DPP4-promoter plasmid. The pGL3-basic luciferase vector without DPP4 promoter was used as a control. The specific effector plasmid of CTCF (pCDH-CTCF) was also constructed. Transfection into 293T cell lines was performed using Lipofectamine 3000 (Invitrogen, USA). Cells were harvested 24 h after transfection to evaluate luciferase activity using a Dual-Luciferase® Reporter 1000 Assay system (Promega, Madison, WI, USA) for detection of the promoter activities. The ratio of Firefly to Renilla luciferase activity was used to express the luciferase activity.

### Culture of Alpha Mouse Liver 12 (AML12) Cells and Establishment of NAFLD Cell Model

The AML12 cells were purchased from the Cell Bank of the Chinese Academy of Sciences (Shanghai, China) and cultivated in Dulbecco's Modified Eagle's Medium (DMEM) (Thermo Fisher Scientific, Inc., Waltham, MA, USA) containing 10% fetal bovine serum (FBS) (GIBCO, Grand Island, NY) at 37°C and 5% CO_2_. The cells of passages 1–3 were used to perform the subsequent experiments. For NAFLD *in vitro* model establishment, AML12 cells were exposed to 250 μM palmitic acid (PA) and 1000 ng/ml lipopolysaccharide (LPS) for 24 h.

### Cell Treatments

After the establishment of the NAFLD *in vitro* model, the cells were processed with different concentrations of gardenoside (10, 25, and 50 μM) for 24 h to determine the optimal concentration of gardenoside for treating the NAFLD *in vitro* model.

To explore the mechanism of therapeutic effects of gardenoside on NAFLD, AML12 cells were divided into five groups and processed with different treatments as follows: control group (AML12 cells cultured in medium without other treatments), NAFLD model group (NAFLD *in vitro* model cultured in medium without other treatments), gardenoside group (NAFLD *in vitro* model cultured in medium containing 50 μM gardenoside), siRNA CTCF group (NAFLD *in vitro* model transfected with CTCF-specific siRNA, and then cultured in medium without other treatment) and its corresponding negative control group (siRNA NC group) (NAFLD *in vitro* model transfected with scrambled siRNA, and then cultured in medium without other treatment), CTCF vector group (NAFLD *in vitro* model transfected with CTCF expression vector and then cultured in medium without other treatments) and its corresponding negative control group (Control vector group) (NAFLD *in vitro* model transfected with empty control vector, and then cultured in medium without other treatments), and siRNA DPP4 group (NAFLD *in vitro* model transfected with DPP4-specific siRNA, and then cultured in medium without other treatment) and its corresponding negative control group (siRNA NC group) (NAFLD *in vitro* model transfected with scrambled siRNA, and then cultured in medium without other treatment).

### Cell Transfection

The CTCF-specific and DPP4-specific siRNAs were used for silencing CTCF and DPP4, respectively. Scrambled siRNAs were used as the negative controls (NC). The siRNA sequences were as follows: CTCF-siRNA#1, 5′-GCCCTCTCTCTTGATCGTAAA-3′; CTCF-siRNA#2, 5′-GGATGGCATGCTACGATCAGT-3′; CTCF-siRNA#3, 5′-GATGGCATGCTACGATCAGTT-3′; DPP4-siRNA#1, 5′-GTTCCCCTTATCCCTCACTCA-3′; DPP4-siRNA#2, 5′-GCCTTGCGCTAACTAATGTTT-3′; DPP4-siRNA#3, 5′-GCTCAAGCTACAGAGTTCAGT-3′; and siRNA NC: 5′-GGATGATTGATGCGGTAAGAA-3′. According to the instructions of the manufacturer, the Lipofectamine® 2000 Transfection Reagent kit (Thermo Fisher Scientific, Inc., Waltham, MA, USA) was used to transfect the AML12 cells in the logarithmic growth phase for 24 h. After determining the transfection efficiency of the siRNAs by quantitative real-time PCR (RT-qPCR) and Western blotting, the siRNA plasmid with the highest silencing efficiency was chosen to transfect the NAFLD model for further experiments. For the overexpression of CTCF, the pLenti-CMV-CTCF vector (CTCF Vector) lentiviral constructs were transduced in the cells. The negative control vector was the empty pLenti-CMV vector (Control Vector).

### Animal and NAFLD Mouse Model Establishment

All experiments and protocols were performed in accordance with the Shanghai University of Traditional Chinese Medicine Animal Ethics and Use Committee. Fifty male C57BL/6J mice (weight: 22–28 g, age: 10 weeks) were obtained from Shanghai Slack Laboratory Animal Co. (Shanghai, China) and fed in a specific pathogen-free (SPF) room. All mice acclimatized in the new environment (a cycle of 12 h light/dark with 23–25°C temperature and 40–70% relative humidity) for a week and were allowed to drink and eat freely. After adaptive feeding, 10 mice were assigned to the control group, which were fed a normal diet. The other 40 mice were used to establish the NAFLD model, in which the normal diet was replaced with a high-fat diet (D12492) (Research diets Inc., New Brunswick, NJ, USA). After a 12 week duration of a high-fat diet, NAFLD mouse models were established and randomly divided into four groups (10 mice per group) as follows: (1) NAFLD model group (NAFLD mice without treatment), (2) gardenoside group [NAFLD mice treated with intragastrical administration of gardenoside (40 mg/kg) in each day at a specific time], (3) siRNA NC group (NAFLD mice with intravenous injection of with empty adenovirus vector every 2 weeks), and (4) siRNA CTCF group (NAFLD mice injected with adenovirus siRNA vector of CTCF). All treatments for NAFLD mice lasted for 4 weeks.

### Sample Collection

After the last treatment, an intraperitoneal injection of pentobarbital sodium was performed to euthanize all mice. Blood samples were acquired for centrifugation at 1,500 × *g* for 15 min at 4°C to collect serum. Meanwhile, liver tissues were quickly dissected on ice. One-half of each sample was conserved in 4% paraformaldehyde (PFA) solution for 24 h at room temperature and embedded in paraffin for slicing into liver sections (4 and 8 μm thicknesses). The remaining tissues for examining mRNA and protein expression were stored at −80°C.

### H&E Staining

As previously described (Andrés-Manzano et al., 2015), the 4 μm liver sections were stained with H&E after dehydration of a graded ethanol series. The staining sections were investigated and analyzed under the OLYMPUS BX53 microscope (Shanghai, China). The histological score of the liver was evaluated quantitatively according to three aspects: hepatocellular ballooning, macrovesicular steatosis, and inflammatory cell infiltration.

### Oil Red O Staining

The neutral triglycerides and lipids of liver tissues and AML12 cells from different groups were observed by staining with Oil red O dye. Briefly, the frozen liver sections (8 μm) and fixed AML12 cells were dyed with Oil red O solution (1%) and washed three times with distilled water. Then, staining sections and cells were differentiated with isopropanol, rinsed two times with distilled water, followed by counterstaining in Mayer's hematoxylin before examination under the OLYMPUS BX53 microscope.

### Biochemical Indexes

An automatic biochemical analyzer (Hitachi7170S, Tokyo, Japan) was applied to detect serum levels of NAFLD-related biochemical indexes, which included total cholesterol (TC), alanine aminotranferease (ALT), and aspartate aminotransferase (AST). The ALT, AST, and TC assay kits were obtained from Nanjing Jiancheng Bioengineering Institute (China) and the procedures followed were according to the protocol of the manufacturer.

### DPP4 Activity

The DPP4 activity was determined by a colorimetric assay on a microplate reader (Molecular Devices, Menlo Park, CA, USA) at a wavelength of 460 nm. The detections were performed in duplicates. After the addition of a 50 μl serum sample in 96-well plates, 10 μl assay buffer was added, and the plate was incubated for 15 min before performing an enzymatic reaction. Next, 2 μl of the substrate and 38 μl of assay buffer were added to start the reaction, and 7-Amino-4-Methyl Coumarin (AMC), released from H-Gly-Pro-AMC that was cleaved by DPP4, was monitored spectrophotometrically every 5 min at 37°C. The activity of DPP4 was expressed as pmol/min/μg.

### ELISA Detection of Cytokines

The ELISA was employed to evaluate levels of IL-1β and IL-18 in serum samples from different groups. The mouse IL-1 beta ELISA kit was procured from Abcam (Cambridge, MA, USA), whereas the mouse IL-18 ELISA kit was bought from Invitrogen (Carlsbad, CA, USA).

### Intracellular ROS Measurement

Levels of intracellular ROS were examined by the use of the fluorescent probe 2′, 7′-dichlorofluorescin diacetate (DCFH-DA) (Sigma-Aldrich, Poznan, Poland). The samples of each group were loaded with DCFH-DA (10mM) at 37°C in the dark and then rinsed three times before observation of intracellular ROS fluorescence under an Olympus BX51 fluorescence microscope (Tokyo, Japan). The fluorescence intensity reflected the generation of intracellular ROS among different groups.

### Intracellular Triglyceride (TG) Analysis

The TG assay kit (Cell Biolabs Inc., San Diego, CA, USA) was applied to measure TG levels of AML12 cells according to the protocol of the manufacturer.

### Cell Counting Kit-8 (CCK8) Assay

The CCK-8 kit was bought from Dojindo Laboratories (Tokyo, Japan) and applied to assess the cell viability of AML12 cells treated with different methods. In brief, after treating cells according to our study design for 24 h, 10 μl CCK-8 reagent was added to each sample. The cells were then conserved in a 5% CO_2_ incubator at 37°C and cultured for another 2 h. The cell viability was calculated based on absorbance that was determined at a wavelength of 450 nm.

### Lactate Dehydrogenase (LDH) Release

To study LDH release, CytoTox 96 Non-Radioactive Cytotoxicity Assay (Promega, USA) was used to determine the LDH content in cell lysates. The reading was taken with a microplate reader at the wavelength of 490 nm. The release of LDH was quantified as the ratio of the values in different groups to the value of the control group.

### Morphometric Analysis on the Ultrastructure of AML12 Cells

A transmission electron microscope was used to observe and image the ultrastructure of treated cells. Briefly, after different treatments, AML12 cells were harvested and fixed with 2.5% glutaraldehyde, then post-fixed in 1% phosphate-buffered osmium tetroxide. Subsequently, the fixed cells were embedded and stained with uranyl acetate as well as lead citrate, and finally imaged and analyzed under a transmission electron microscope (JEOL, Tokyo, Japan).

### Hoechst 33342/Propidium Iodide (PI) Fluorescent Staining

The fluorescent dyes, which included Hoechst 33342 and PI, were used to double stain AML12 cells for the evaluation of pyroptosis. The staining assay for the cell was performed as described previously (Yu et al., 2019). After interventions, 5 g/L Hoechst 33342 and 10 g/L PI (Beyotime, Shanghai, China) were used to stain the AML12 cells at 25°C for 10 min in the dark. Finally, the stained cells were assessed for apoptosis under an Olympus BX51 fluorescence microscope.

### Immunofluorescence Staining

For protein (NLRP3, caspase-1 p20) localization within cells and to visualize changes in their expression levels *in situ*, AML12 cells were stained by immunofluorescence. After different treatments, cells were rinsed and fixed, followed by incubation with 1% bovine serum albumin (BSA) for finishing a blocking step. Then, the cells were reacted with primary antibodies (anti-NLRP3 and anti-caspase-1 p20, respectively) (Abcam, UK) for overnight reaction at 4°C, followed by conjugation with secondary antibodies at 37°C for 1 h in the dark. After counterstaining 4, 6-diamidino 2-phenylindole (DAPI) with cells, the staining of the cells was recorded by Olympus BX51 fluorescence microscope.

### Quantitative Real-Time PCR (RT-qPCR)

By using the TRIzol reagent, total RNA was respectively extracted from AML12 cells and liver tissues of each group. The Reverse Transcription kit (Life Technologies, Carlsbad, CA, USA) was used to reverse transcribe RNA into cDNA. Afterward, the specific cDNA was subsequently used to perform a qPCR reaction. In this study, β-actin was used as the internal reference to calculate the relative expression of studied genes based on the 2^−Δ*ΔCT*^ approach (Livak and Schmittgen, 2001). The primer sequences for RT-qPCR used in this study were as follows: β-actin forward (F): 5′-TCCCTGAGACGCTAGATGAGG-3′, reverse (R): 5′-CGTTTAGCAGTTTTGTCAGCTC-3′; CTCF F: 5′-GAACGAACTAGGCTCAAGAGAG-3′, R: 5′-GTGGGCTTCCGGAATAGCTTCC-3′; DPP4 F: 5′-GGGTCACATGGTCACCAGTG-3′, R: 5′-TCTGTGTCGTTAAATTGGGCATA-3′; NLRP3 F: 5′-GAGTTCTTCGCTGCTATGT-3′, R: 5′-ACCTTCACGTCTCGGTTC-3′; ASC F: 5′-CTCTGTATGGCAATGTGCTGAC-3′, R: 5′-GAACAAGTTCTTGCAGGTCAG-3′; CTGF F: 5′-AGAACTGTGTACGGAGCGTG-3′, R: 5′-AAAACGGAGTGACTAGGGGC-3′; Collagen IV: F: 5′-ATGGCTTGCCTGGAGAGATAGG-3′, R: 5′-TGGTTGCCCTTTGAGTCCTGGA-3′; Collagen V F: 5′-AGATGGCATCCGAGGTCTGAAG-3′, R: 5′-GACCTTCAGGACCATCTTCTCC-3′; ASC F: 5′-CTGCTCAGAGTACAGCCAGAAC-3′, R: 5′-CTGTCCTTCAGTCAGCACACTG-3′.

### Western Blot

Relative protein expression levels were analyzed using Western blot. Briefly, AML12 cells and liver tissues were separately processed with radioimmunoprecipitation assay (RIPA) lysis buffer for obtaining total proteins which were further quantified with the BCA Protein Assay Kit (Thermo Scientific, Waltham, MA, USA). The harvested proteins were separated by SDS-PAGE before transfer onto 0.45 mm polyvinylidene fluoride (PVDF) membranes. Then, the membranes were blocked to prevent unspecific binding and maintained in primary antibody solutions overnight (4°C). Incubation with secondary antibodies was thereafter performed at a temperature of 25°C, ensued by washing steps. The visualization of studied proteins was performed by use of ECL™ Western Blotting Detection reagents (Amersham Pharmacia Biotech UK Ltd., Buckinghamshire, UK), and analyzed by Image J. The primary antibodies were obtained from Abcam (UK) and showed as follows: CTCF (1:1,000), DPP4 (1:1,000), NLRP3 (1:800), ASC (1:1,000), GSDMD-N (1:1,000), pro-Caspase-1 (1:1,000), Caspase-1-p20 (1:1,000), pro-IL-1β (1:1,000), IL-1β (1:800), and GAPDH (1:5,000).

### Flow Cytometry

Cell apoptosis and pyroptosis detection was achieved by flow cytometry using the BD FACS Canto II cytometer (BD Biosciences, San Diego, CA, USA). The FAM-FLICA®Caspase Assays (Immunochemistry Technologies, Minneapolis, MN) were achieved by direct supplementation of the FLICA probes to 100 μl of cells (1 × 10^6^ cells/ml). After incubating the mixture in the dark for 1 h in a 5% CO_2_ incubator at 37°C, a solution of PBS added with 2 mM EDTA was used for 3 washes to remove the reagent and keep FLICA probes covalently bound to their targets. Next, we used PI for 30 mins of staining, followed by flow cytometry analysis.

### Statistical Analysis

All statistical analyses were conducted on version 9.0.0 of GraphPad Prism (Graphpad Software, Inc., La Jolla, USA). All experiments were repeated at least three times, and all data were presented as mean ± SD. In the present study, a one-way ANOVA with a *post-hoc* test (Bonferroni) was used to analyze the difference among the groups. A *p*-value of < 0.05 showed that the discrepancy was statistically significant.

## Results

### CTCF Is a Positive Regulator of DPP4

**Figure 1:**
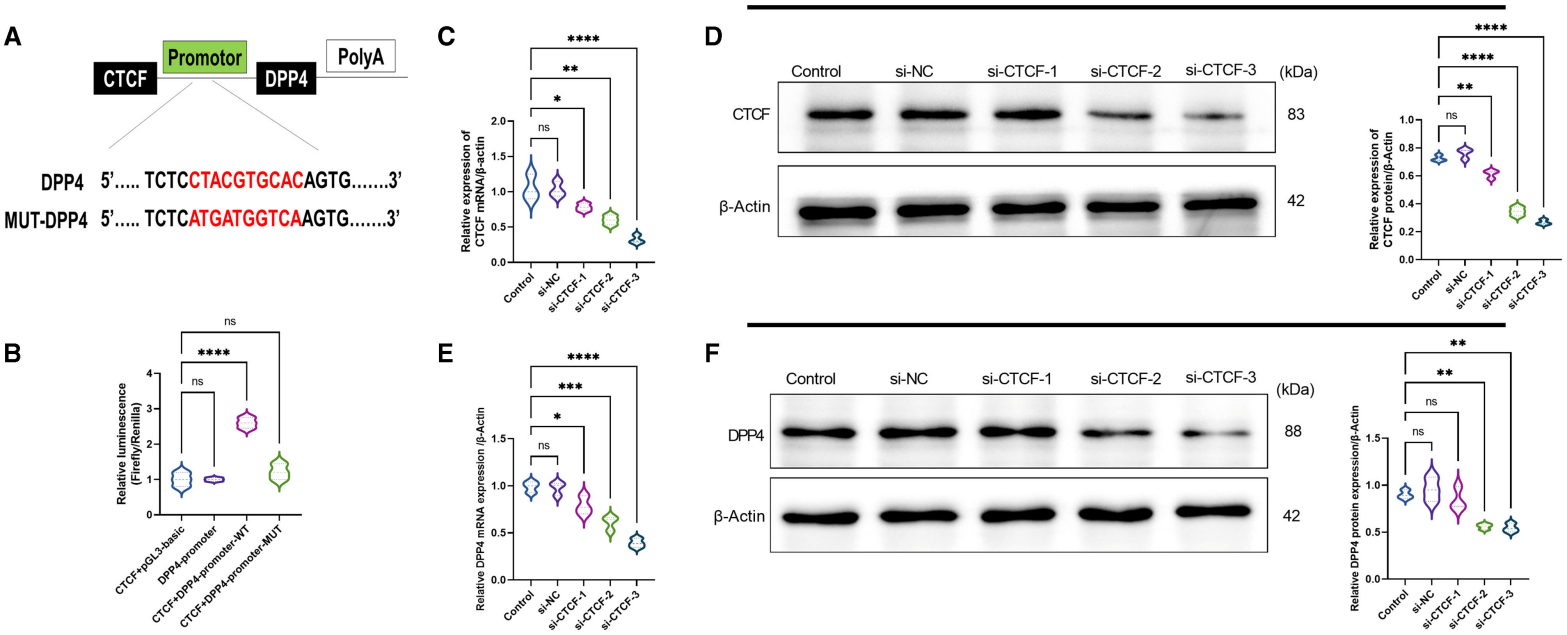
CCCTC-binding factor (CTCF) is a positive regulator of dipeptidyl peptidase-4 (DPP4). **(A)** Prediction of the targeted binding site between the promoter region of the DPP4 gene and the transcription factor CTCF by MEME suite and JASPAR databases. **(B)** The predicted binding site between the promoter region of the DPP4 gene and the transcription factor CTCF was verified using a dual-luciferase reporter assay. **(C)** The relative mRNA expression of CTCF in the untransfected, si-NC, and different si-RNAs of CTCF (si-CTCF) were detected by RT-qPCR. **(D)** The relative protein expression of CTCF in the untransfected, si-NC, si-CTCF-1, si-CTCF-2, and si-CTCF-3 groups were measured by Western blot. **(E)** The relative mRNA expression of DPP4 in the untransfected, si-NC, si-CTCF-1, si-CTCF-2, and si-CTCF-3 groups were detected by RT-qPCR. **(F)** The relative protein expression of DPP4 in the untransfected, si-NC, si-CTCF-1, si-CTCF-2, and si-CTCF-3 groups were measured by Western blot. ns, non-significant, ^*^p < 0.05, ^**^p < 0.01, ^***^p < 0.001, and ^****^p < 0.0001 in the comparison between the indicated groups.

**Figure 2:**
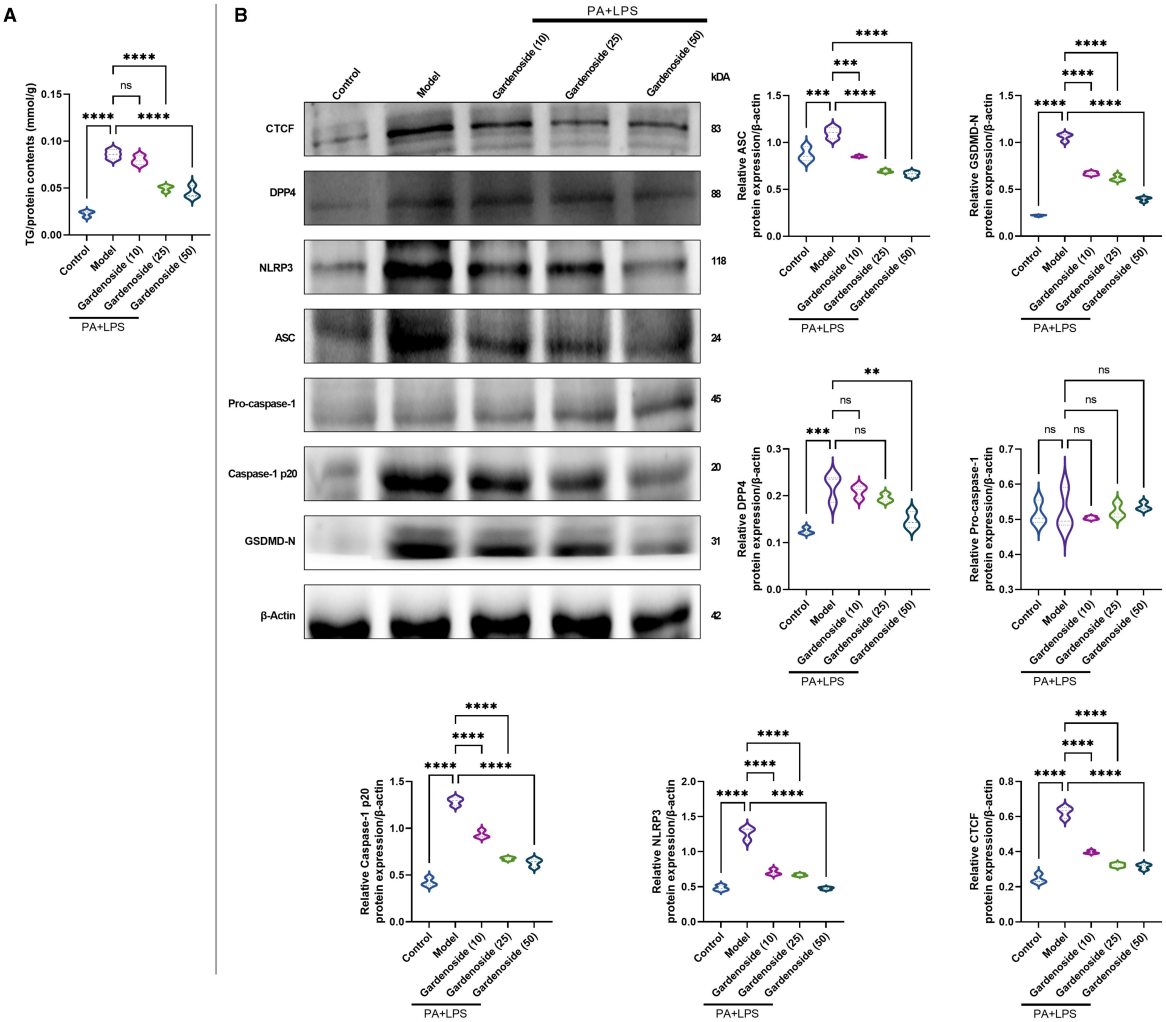
Gardenoside reduced the accumulation of lipid and pyrin-domain containing 3 (NLRP3) inflammasome and pyroptosis-associated proteins in hepatocytes. **(A)** Detection of triglyceride (TG) content in alpha mouse liver (AML) cells of control, model, and gardenoside (10, 25, and 50 μM) groups. **(B)** Screening of the optimal concentration of gardenoside to inhibit the activation of NLRP3 inflammasome and hepatocyte pyroptosis by Western blot. Violin plots were used to depict the relative protein expression levels of CTCF, DPP4, NLPR3, apoptosis-related speck-like protein (ASC), pro-caspase-1 p20, caspase-1 p20, GSDMD-N, pro-IL-1β, and IL-1β in AML cells of control, cell NAFLD model, and gardenoside (10, 25, and 50 μM) groups by Western blot. ns, non-significant, **p < 0.01, ***p < 0.001, and ****p < 0.0001 in the comparison between the indicated groups.

To explore whether there is a regulatory relationship between CTCF and DPP4, MEME suite and JASPAR database were used to predict the targeted binding site of CTCF and DPP4. It was found that there was a highly matched binding site with 10 base pairs between the promoter region of the DPP4 gene and the transcription factor CTCF (Figure 2A). Subsequently, their interaction was verified in AML12 cells using a dual-luciferase reporter assay. The results showed that compared with co-transfection with plasmids pCDH-CTCF and pGL3-basic, or transfection with pGL3-DPP4-promoter alone, the cotransfection with plasmids pCDH-CTCF and pGL3-DPP4-promoter-WT led to significantly increased relative luciferase activity (*p* < 0.01) (Figure 2B). However, the relative luciferase activity of the DPP4 promoter mutant group was lower than that of the WT group (*p* < 0.01) and did not significantly change compared with the CTCF + pGL3-basic vector or the DPP4 promoter alone groups (Figure 2B). As shown in Figures 1C,D, to study the molecular regulation mechanism of CTCF in the pathological process of NAFLD, we designed three siRNAs targeting CTCF and used RT-qPCR and Western blot to detect the mRNA and protein expression levels of CTCF and DPP4, respectively. When compared with the untransfected and si-NC groups, the mRNA and protein expression levels of CTCF in the si-CTCF-1, si-CTCF-2, and si-CTCF-3 groups were significantly downregulated, especially in the siRNA3 group (Figures 1C,D). The same tendency was recorded regarding the mRNA and protein expression levels of DPP4, confirming that CTCF is a positive regulator of DPP4 (Figures 1E,F). The si-CTCF-3 was selected for downstream experiments.

### Gardenoside Reduces the Accumulation of Lipid in Hepatocytes

To explore the hepatoprotective mechanism of gardenoside and its influence on the regulation of inflammasome and pyroptosis in NAFLD, we preliminarily examined the influence of gradient concentrations of gardenoside on lipid accumulation and the expression of proteins involved in the stimulation of NLRP3 inflammasome and caspase-1-induced pyroptosis. The TG assay *in vitro* showed that both 25 and 50 μM gardenoside could significantly reduce PA+LPS-induced lipid accumulation in the AML12 cell line (*p* < 0.01), and the lipid-lowering effect of gardenoside was concentration-dependent (Figure 2A). In addition, 25 and 50 μM gardenoside could significantly downregulate the protein expression of CTCF, DPP4, and PRRs (NLRP3, ASC), but promoted the expression of downstream GSDMD precursors and activated caspase-1 p20 (Figure 2B). These preliminary results hinted that gardenoside is a potential anti-NAFLD drug and its mechanism of action may involve inflammasome and pyroptosis regulated by the CTCF/DPP4 axis, which was aimed to be clarified in the subsequent experiments, where 50 μM gardenoside was used.

### Gardenoside and CTCF Silencing Inhibits the Activation of NLRP3 Inflammasome and Caspase-1-Induced Pyroptosis in Hepatocytes

**Figure 3:**
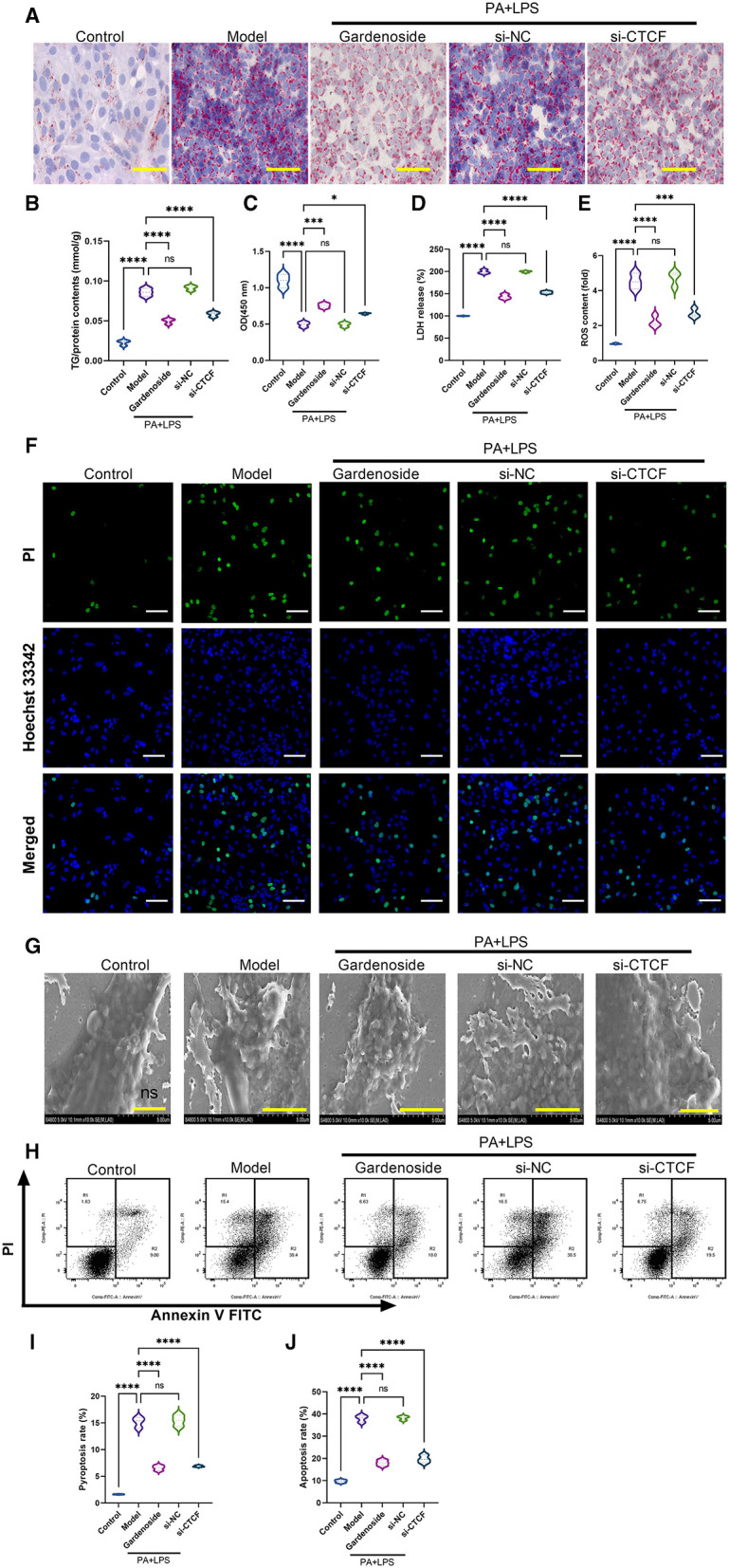
Gardenoside and CTCF silencing inhibited lipid accumulation, intracellular reactive oxygen species (ROS), LDH release, apoptosis, and pyroptosis in the cell model of NAFLD. **(A)** Oil Red O staining showing lipid accumulation level in AML cells of control, model, gardenoside, si-NC, and si-CTCF groups (Scale bar: 50μm). **(B)** Detection of the TG content in AML cells of control, model, gardenoside, si-NC, and si-CTCF groups. **(C)** Detection of cell proliferation in AML cells of control, model, gardenoside, si-NC, and si-CTCF groups. **(D)** Detection of LDH release in AML cells of control, model, gardenoside, si-NC, and si-CTCF groups. **(E)** Detection of intracellular ROS production in AML cells of control, model, gardenoside, si-NC, and si-CTCF groups. **(F)** Detection of cell apoptosis by Hoechst/PI staining of AML cells of control, model, gardenoside, si-NC, and si-CTCF groups (scale bar: 50μm). **(G)** Representative transmission electron micrograph of AML cells in different treatment groups (scale bar: 2 μm). **(H)** Representative flow cytometry images indicating the levels of apoptosis and pyroptosis of AML cells in different treatment groups. **(J)** Quantitative representation of pyroptosis rate of AML cells in different treatment groups as detected by flow cytometry. **(J)** Quantitative representation of apoptosis rate of AML cells in different treatment groups as detected by flow cytometry. ns, non-significant, *p < 0.05, ***p < 0.001, and ****p < 0.0001 in the comparison between the indicated groups.

To investigate the role of CTCF/DPP4 in NAFLD-associated inflammasome and pyroptosis, we perfomed a series of experiments after the silencing of CTCF. As shown in Figure 3A, Oil Red O staining indicated a lipid-lowering effect of si-CTCF similar to that of 50 μM gardenoside treatment (Figure 3A). Compared with normal cells in the control group, the AML12 cells of the NAFLD model contained a large number of lipid droplets stained with Oil Red, while gardenoside (50 μM) and CTCF silencing treatment significantly reduced the accumulation of lipids in hepatocytes (Figure 3A). Moreover, the TG analysis confirmed the lipid-lowering effects of both gardenoside and si-CTCF as reflected by the decrease of TG in these groups (Figure 3B), indicating that gardenoside, as well as si-CTCF, may exert hepatoprotective effects in NAFLD. As shown in Figure 3C, in the AML12 cell model of NAFLD, cell viability was significantly reduced, but this decreasing trend was reversed in the cells of gardenoside or si-CTCF treatment groups. In addition, the induction of PA+LPS promoted the generation of LDH (Figure 3D) and intracellular ROS (Figure 3E) in AML12 cells, and gardenoside or CTCF silencing could effectively reverse the upregulation trend (Figures 3D,E). Similar results were obtained in Hoechst 33342/PI fluorescent staining. Compared with the NAFLD model group, the number of apoptotic cells decreased significantly in gardenoside and si-CTCF treatment groups (Figure 3F). Through the observation of transmission electron microscope, we found that compared with the cells in the control group, the cell membrane integrity of hepatocytes in the NAFLD model was impaired, but the pyroptotic process was inhibited by the treatment of gardenoside or CTCF silencing (Figure 3G). The flow cytometry analysis also indicated the increase of both apoptosis and pyroptosis cells in the PA+LPS cells, but this was reversed by gardenoside and si-CTCF (Figures 3H–K).

**Figure 4:**
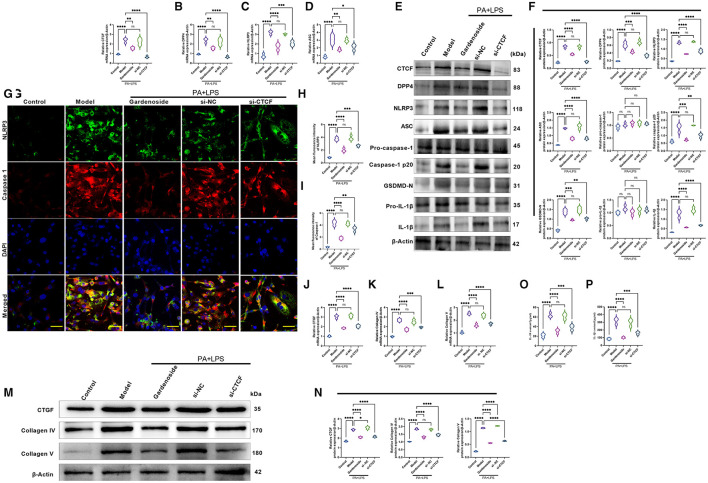
Gardenoside and CTCF silencing inhibited the activation of NLRP3 inflammasome, caspase1-induced pyroptosis, and fibrosis pathways in hepatocytes. **(A)** RT-qPCR detection of the relative mRNA expression of CTCF in AML cells in indicated groups. **(B)** RT-PCR detection of the relative mRNA expression of DPP4 in AML cells in indicated groups. **(C)** RT-PCR detection of the relative mRNA expression of NLRP3 in AML cells in indicated groups. **(D)** RT-PCR detection of the relative mRNA expression of ASC in AML cells in indicated groups. **(E)** Representative bands of Western blot detection of the relative protein expression of CTCF, DPP4, NLRP3, ASC pro-caspase-1 p20, caspase-1 p20, GSDMD-N, pro-IL-1β, and IL-1β in AML cells in indicated groups. **(F)** Densitometry analysis of bands of Western blot detection of the relative protein expression of CTCF, DPP4, NLRP3, ASC pro-caspase-1 p20, caspase-1 p20, GSDMD-N, pro-IL-1β, and IL-1β in AML cells in indicated groups. **(G)** Representative images of immunofluorescence analysis of NLRP3 and Caspase-1 expression in AML cells in indicated groups (scale bar: 50μm). **(H)** Quantification of immunofluorescence intensity of NLRP3 expression in different groups as determined by immunofluorescence analysis. **(I)** Quantification of immunofluorescence intensity of caspase-1 expression in different groups as determined by immunofluorescence analysis. **(J)** RT-qPCR detection of the relative mRNA expression of the fibrosis marker CTGF in AML cells in indicated groups. **(K)** RT-qPCR detection of the relative mRNA expression of the fibrosis marker Collagen IV in AML cells in indicated groups. **(L)** RT-qPCR detection of the relative mRNA expression of the fibrosis marker Collagen V in AML cells in indicated groups. **(M)** Bands of Western blot detection of the relative protein expression of the fibrosis markers CTGF, Collagen IV and Collagen V in AML cells in indicated groups. **(N)** Densitometry analysis of bands of Western blot detection of the relative protein expression of the fibrosis markers CTGF, Collagen IV and Collagen V in AML cells in indicated groups. **(O)** ELISA detection of the protein level of IL-18 in indicated groups. **(P)** ELISA detection of the protein level of IL-1β in indicated groups. ns, non-significant, *p < 0.05, **p < 0.01, ***p < 0.001, and ****p < 0.0001 in the comparison between the indicated groups.

Consistently, RT-qPCR results (Figures 4A–D) revealed that the expression levels of CTCF, DPP4, NLRP3, ASC were significantly upregulated in PA+LPS-treated AML12 cells while Western blot results (Figures 4E,F) indicated that in addition to CTCF, DPP4, NLRP3, and ASC, the downstream protein expression levels of caspase-1 p20, IL-1β and GSDMD-N were increased as well, but gardenoside or CTCF silencing downregulated the mRNA and protein expression levels of these genes (Figures 4A–F). Moreover, the regulatory effects of gardenoside and CTCF silencing on NLRP3 and caspase-1 p20 were confirmed in the results of immunofluorescence (Figures 4G,I). Moreover, the analysis of the expression of fibrosis-related markers by qRT-PCR (Figures 4J–L) and Western blotting (Figures 4M,N) indicated that the expression levels of CTGF, Collagen IV, and Collagen V were all upregulated in the PA+LPS cells but decreased by gardenoside or si-CTCF. The ELISA test also indicated that the inflammatory markers IL-18 and IL-1β were all activated in the PA+LPS-induced cells but inhibited by the gardenoside and si-CTCF treatments (Figures 4O,P).

### CTCF Overexpression Counteracted Gardenoside Inhibition of NLRP3 Inflammasome and Caspase-1-Induced Pyroptosis and Downregulated DPP4 in Hepatocytes

**Figure 5:**
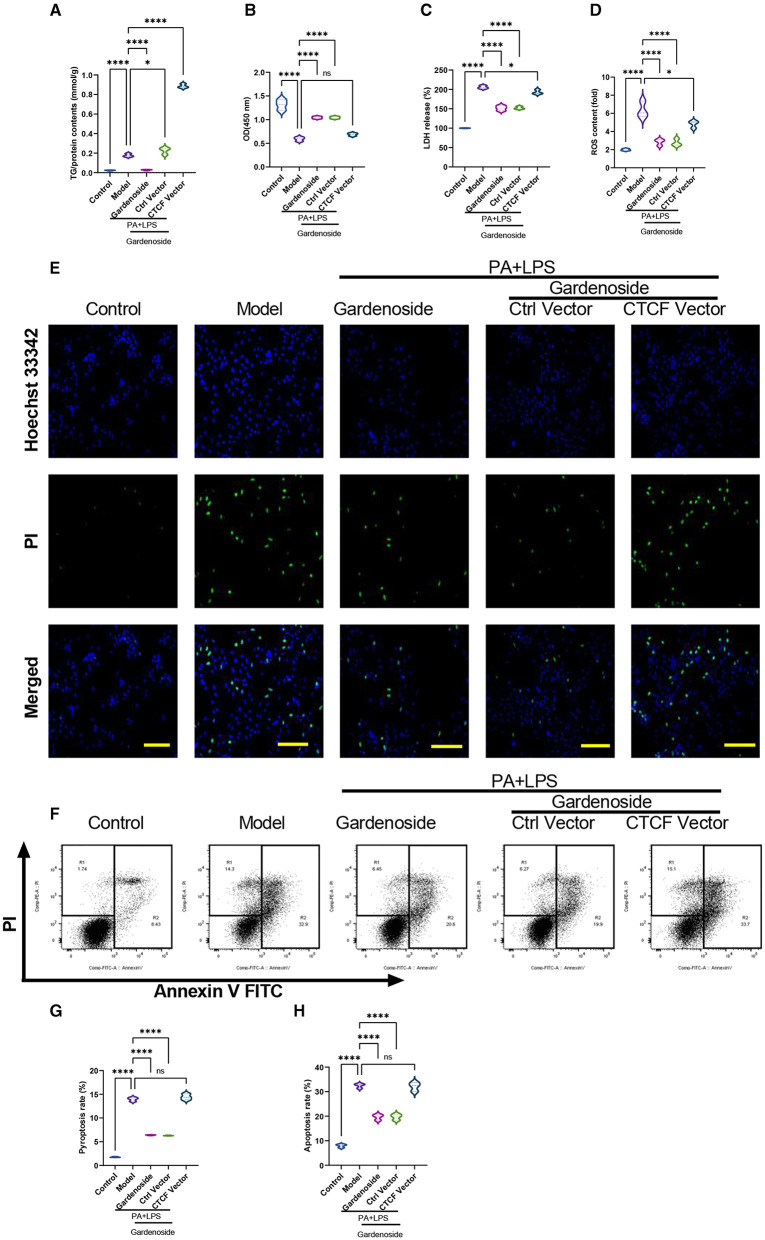
CTCF overexpression counteracted the effect of gardenoside on lipid accumulation, intracellular ROS, LDH release, apoptosis and pyroptosis in the cell model of NAFLD. **(A)** Detection of the TG content in AML cells of indicated groups. **(B)** Detection of cell proliferation in AML cells of indicated groups. **(C)** Detection of LDH release in AML cells of indicated groups. **(D)** Detection of intracellular ROS production in AML cells of indicated groups. **(E)** Detection of cell apoptosis by Hoechst/PI staining of AML cells of indicated groups (scale bar: 50μm). **(F)** Representative flow cytometry images indicating the levels of apoptosis and pyroptosis of AML cells in different treatment groups. **(G)** Quantitative representation of pyroptosis rate of AML cells in different treatment groups as detected by flow cytometry. **(H)** Quantitative representation of apoptosis rate of AML cells in different treatment groups as detected by flow cytometry. ns, non-significant, *p < 0.05, and ****p < 0.0001 in the comparison between the indicated groups.

**Figure 6:**
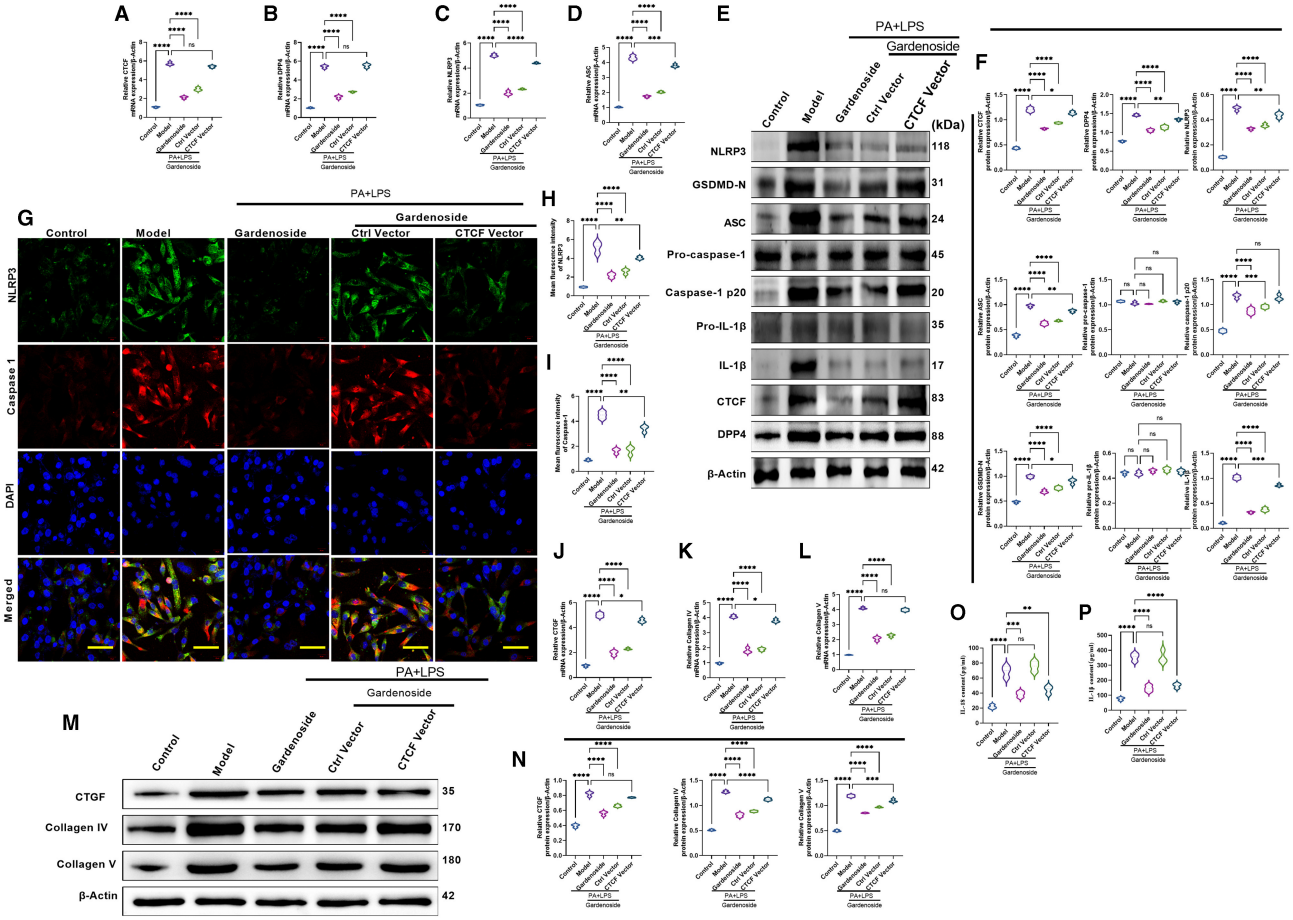
CTCF overexpression counteracted the effect of gardenoside on the activation of the NLRP3 inflammasome, caspase-1-induced pyroptosis and fibrosis pathways in hepatocytes. **(A)** RT-qPCR detection of the relative mRNA expression of CTCF in AML cells in indicated groups. **(B)** RT-PCR detection of the relative mRNA expression of DPP4 in AML cells in indicated groups. **(C)** RT-PCR detection of the relative mRNA expression of NLRP3 in AML cells in indicated groups. **(D)** RT-PCR detection of the relative mRNA expression of ASC in AML cells in indicated groups. **(E)** Representative bands of Western blot detection of the relative protein expression of CTCF, DPP4, NLRP3, ASC pro-caspase-1 p20, caspase-1 p20, GSDMD-N, pro-IL-1β, and IL-1β in AML cells in indicated groups. **(F)** Densitometry analysis of bands of Western blot detection of the relative protein expression of CTCF, DPP4, NLRP3, ASC pro-caspase-1 p20, caspase-1 p20, GSDMD-N, pro-IL-1β, and IL-1β in AML cells in indicated groups. **(G)** Representative images of immunofluorescence analysis of NLRP3 and Caspase-1 expression in AML cells in indicated groups (scale bar: 50 μm). **(H)** Quantification of immunofluorescence intensity of NLRP3 expression in different groups as determined by immunofluorescence analysis. **(I)** Quantification of immunofluorescence intensity of Caspase-1 expression in different groups as determined by immunofluorescence analysis. **(J)** RT-qPCR detection of the relative mRNA expression of the fibrosis marker CTGF in AML cells in indicated groups. **(K)** RT-qPCR detection of the relative mRNA expression of the fibrosis marker Collagen IV in AML cells in indicated groups. **(L)** RT-qPCR detection of the relative mRNA expression of the fibrosis marker Collagen V in AML cells in indicated groups. **(M)** Bands of Western blot detection of the relative protein expression of the fibrosis markers CTGF, Collagen IV, and Collagen V in AML cells in indicated groups. **(N)** Densitometry analysis of bands of Western blot detection of the relative protein expression of the fibrosis markers CTGF, Collagen IV, and Collagen V in AML cells in indicated groups. **(O)** ELISA detection of the protein level of IL-18 in indicated groups. **(P)** ELISA detection of the protein level of IL-1β in indicated groups. ns, non-significant, *p < 0.05, **p < 0.01, ***p < 0.001, and ****p < 0.0001 in the comparison between the indicated groups.

To confirm the effect of CTCF on the biological processes involved in NAFLD and its correlation with gardenoside, we proceeded to the overexpression of CTCF and treated the cells with gardenoside. Contrary to the CTCF silencing, CTCF overexpression reversed the TG content (Figure 5A) and cell proliferation increase induced by gardenoside (Figure 5B) and counteracted the effects of gardenoside on LDH release (Figure 5C), intracellular ROS content (Figure 5D), and cell pyroptosis and apoptosis (Figures 5E,H). As shown in Figures 6A–F, CTCF overexpression counteracted the effect of gadenoside on the expression levels of NLRP3, GSDMD, Caspase-1 p20, IL-1β, CTCF, and DPP4 in the NAFLD model. Immunofluorescence also indicated that the expression levels of NLRP3 and caspase-1 were inhibited by gardenoside in the NAFLD cell model, but reversed by CTCF overexpression (Figures 6G–I). The same counteracting effects of CTCF overexpression on the expression of fibrosis markers (Figures 6J–N) and inflammatory markers (Figures 6O,P) were also recorded. These results suggested that gardenoside may inhibit the activation of NLRP3 inflammasome and caspase1-induced pyroptosis via the CTCF/DPP4 signaling pathway.

### Silencing of DPP4 Mimicked the Inhibitory Effect of Gardenoside and CTCF Silencing on NLRP3 Inflammasome and Caspase-1-Induced Pyroptosis in Hepatocytes

**Figure 7:**
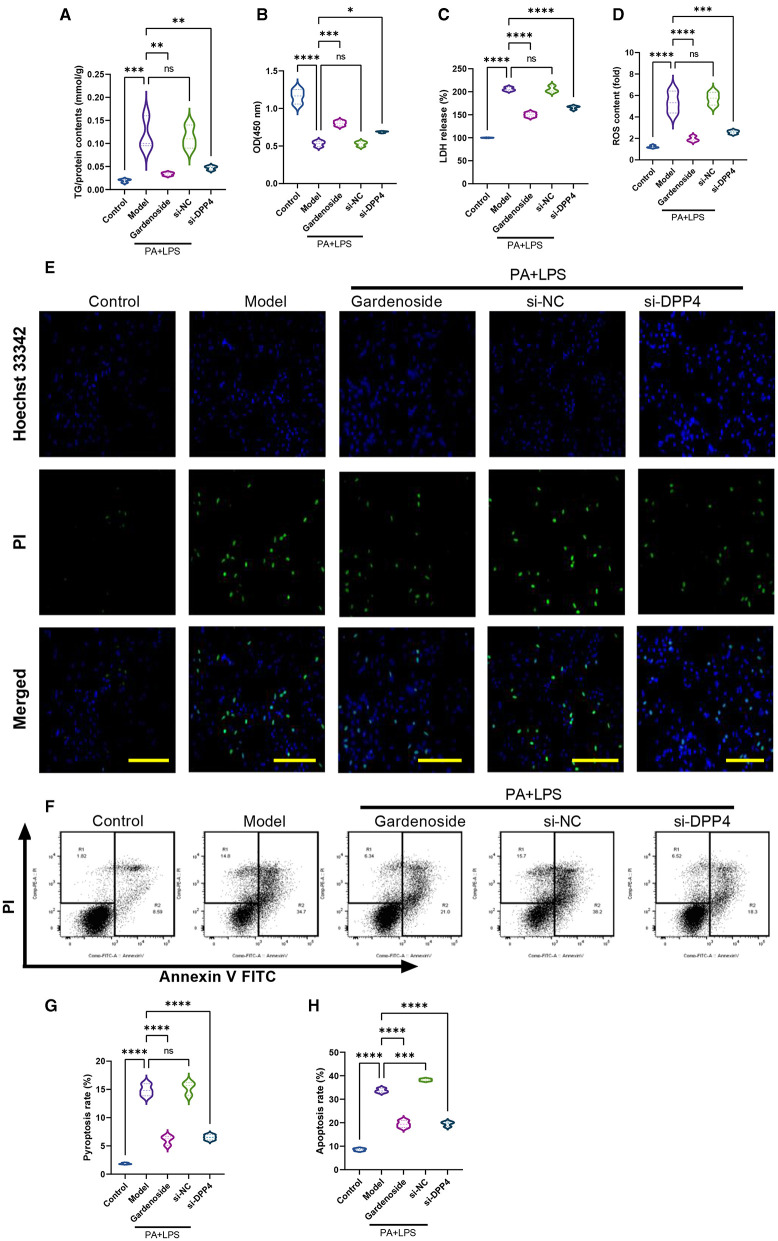
DPP4 silencing mimicked the effect of gardenoside and CTCF silencing on lipid accumulation, intracellular ROS, LDH release, apoptosis and pyroptosis in the cell model of NAFLD. **(A)** Detection of the TG content in AML cells of indicated groups. **(B)** Detection of cell proliferation in AML cells of indicated groups. **(C)** Detection of LDH release in AML cells of indicated groups. **(D)** Detection of intracellular ROS production in AML cells of indicated groups. **(E)** Detection of cell apoptosis by Hoechst/PI staining of AML cells of indicated groups (scale bar: 50 μm). **(F)** Representative flow cytometry images indicating the levels of apoptosis and pyroptosis of AML cells in different treatment groups. **(G)** Quantitative representation of pyroptosis rate of AML cells in different treatment groups as detected by flow cytometry. **(H)** Quantitative representation of apoptosis rate of AML cells in different treatment groups as detected by flow cytometry. ns, non-significant, *p < 0.05, **p < 0.01, ***p < 0.001, and ****p < 0.0001 in the comparison between the indicated groups.

**Figure 8:**
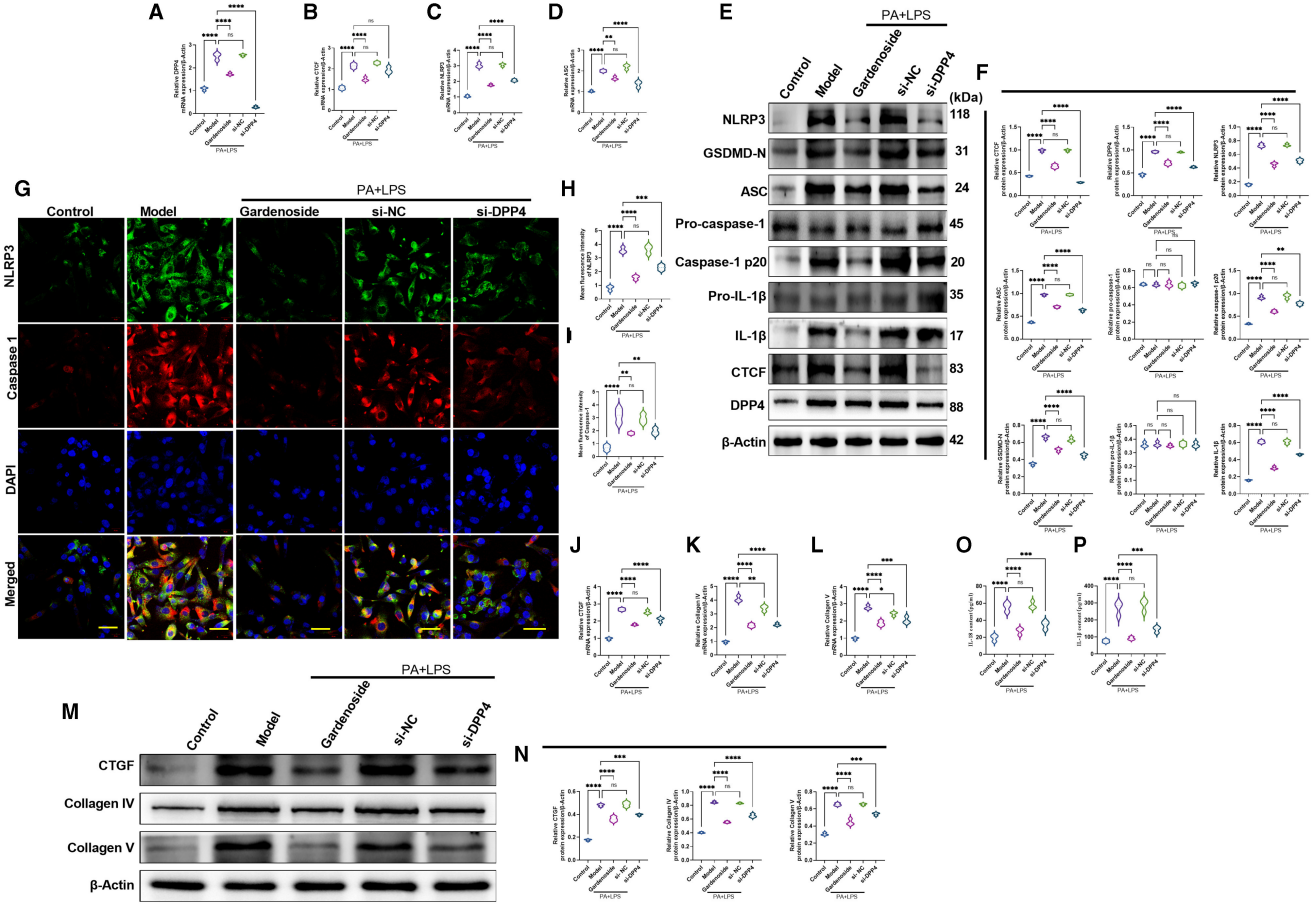
DPP4 silencing mimicked the effect of gardenoside on the activation of the NLRP3 inflammasome, caspase1-induced pyroptosis and fibrosis pathways in hepatocytes. **(A)** RT-qPCR detection of the relative mRNA expression of DPP4 in AML cells in indicated groups. **(B)** RT-PCR detection of the relative mRNA expression of CTCF in AML cells in indicated groups. **(C)** RT-PCR detection of the relative mRNA expression of NLRP3 in AML cells in indicated groups. **(D)** RT-PCR detection of the relative mRNA expression of ASC in AML cells in indicated groups. **(E)** Representative bands of Western blot detection of the relative protein expression of CTCF, DPP4, NLRP3, ASC pro-caspase-1 p20, caspase-1 p20, GSDMD-N, pro-IL-1β, and IL-1β in AML cells in indicated groups. **(F)** Densitometry analysis of bands of Western blot detection of the relative protein expression of CTCF, DPP4, NLRP3, ASC pro-caspase-1 p20, caspase-1 p20, GSDMD-N, pro-IL-1β, and IL-1β in AML cells in indicated groups. **(G)** Representative images of immunofluorescence analysis of NLRP3 and Caspase-1 expression in AML cells in indicated groups (Scale bar: 50 μm). **(H)** Quantification of immunofluorescence intensity of NLRP3 expression in different groups as determined by immunofluorescence analysis. **(I)** Quantification of immunofluorescence intensity of Caspase-1 expression in different groups as determined by immunofluorescence analysis. **(J)** RT-qPCR detection of the relative mRNA expression of the fibrosis marker CTGF in AML cells in indicated groups. **(K)** RT-qPCR detection of the relative mRNA expression of the fibrosis marker Collagen IV in AML cells in indicated groups. **(L)** RT-qPCR detection of the relative mRNA expression of the fibrosis marker Collagen V in AML cells in indicated groups. **(M)** Bands of Western blot detection of the relative protein expression of the fibrosis markers CTGF, Collagen IV, and Collagen V in AML cells in indicated groups. **(N)** Densitometry analysis of bands of Western blot detection of the relative protein expression of the fibrosis markers CTGF, Collagen IV, and Collagen V in AML cells in indicated groups. **(O)** ELISA detection of the protein level of IL-18 in indicated groups. **(P)** ELISA detection of the protein level of IL-1β in indicated groups. ns, non-significant, *p < 0.05, **p < 0.01, ***p < 0.001, and ****p < 0.0001 in the comparison between the indicated groups.

To confirm the hypothesis that gardenoside inhibits the activation of NLRP3 inflammasome and caspase-1-induced pyroptosis *via* the CTCF/DPP4 signaling pathway, the expression of DPP4 was silenced and tested its effect on different biological processes. Silencing of DPP4 mimicked the effect of CTCF silencing on TG content (Figure 7A), cell proliferation (Figure 7B), LDH release (Figure 7C) pyroptosis, and intracellular ROS production (Figure 7D). The Hoechst 33342/PI staining also indicated that similar to si-CTCF and gardenoside, si-DPP4 decreased cell apoptosis in the NAFLD cell model (Figure 7E). The flow cytometry experiment also showed the mimicking effect of CTCF and gardenoside by DPP4 on cell pyroptosis and apoptosis (Figures 7F–H). Silencing of DPP4 was followed by decreased mRNA expression of DPP4, CTCF, NLRP3, and ASC in the NAFLD cell model, which mimicked the effect of gardenoside and si-CTCF (Figures 8A–D). In Western blot analysis, the expression levels of CTCF, DPP4, NLRP3, ASC, caspase-1 p20, IL-1β, and GSDMD-N in the NAFLD cell model were also decreased by si-DPP4, which conformed with the effect of si-CTCF and gardenoside (Figures 8E,F). Furthermore, immunofluorescence assay showed that the effect of si-DPP4 on the expression of caspase1 and NLRP3 was similar to the effects of both gardenoside and si-DPP4 (Figures 8G–I). Similarly, DPP4 mimicked the effect of CTCF silencing on the expression of fibrosis markers at mRNA (Figures 8J–L) and protein (Figures 8M,N) levels. Moreover, si-DPP4 mimicked the effect of gardenoside and si-CTCF on the levels of IL-18 and IL-1β (Figures 8O,P). These results confirmed that gardenoside inhibits the activation of NLRP3 inflammasome and caspase1-induced pyroptosis by inhibiting the CTCF/DPP4 signaling pathway.

### Gardenoside Inhibits NLRP3 Inflammasome and Caspase1-Induced Pyroptosis *via* Inhibiting CTCF/DPP4 Signaling in NAFLD Mouse Model

**Figure 9:**
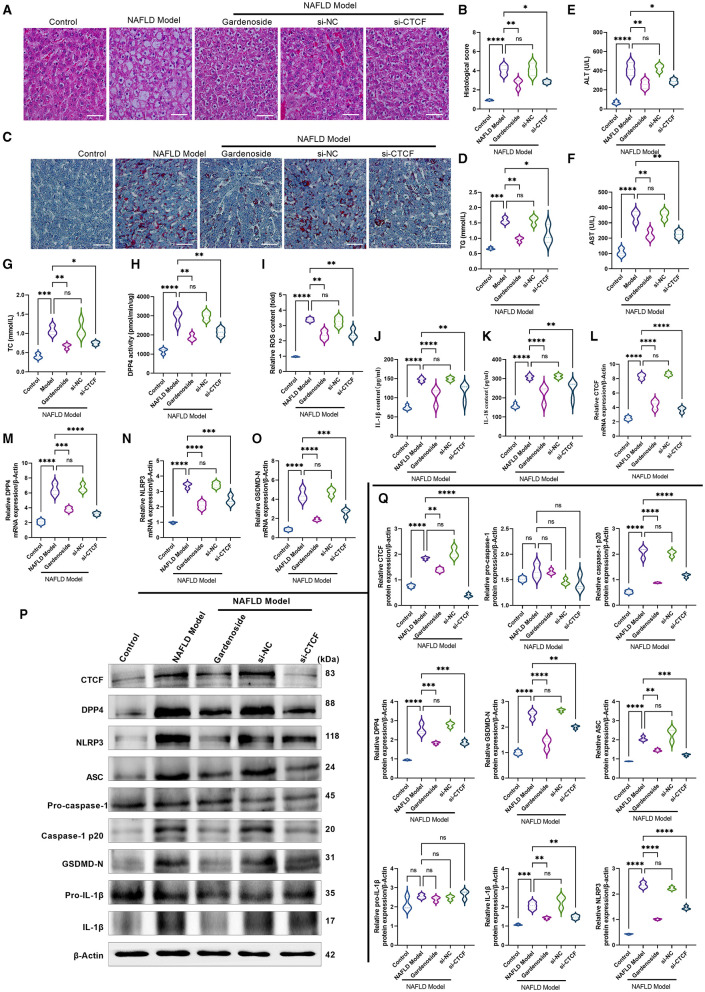
Gardenoside inhibits the lipid accumulation and hepatopathy by the activation of NLRP3 inflammasome and caspase1-induced pyroptosis via the CTCF/DPP4 axis in the NAFLD mice model. **(A)** HE staining (Scale bar: 100 μm), **(B)** histological score, and **(C)** Oil red O staining (Scale bar: 100μm) of liver tissue sections in different groups. Serum levels of **(D)** TG, **(E)** ALT, **(F)** AST, and **(G)** TC in different treatment groups. **(H)** DPP4 activity in serum detected by colorimetric assay. **(I)** Levels of intracellular ROS are determined by the DCFH-DA probe. **(J)** Levels of IL-1β are determined by ELISA. **(K)** Levels of IL-18 are determined by ELISA. **(L)** The expression of CTCF mRNA is evaluated by RT-PCR. **(M)** The expression of DPP4 mRNA is evaluated by RT-PCR. **(N)** The expression of NLRP3 mRNA is evaluated by RT-PCR. **(O)** The expression of ASC mRNA is evaluated by RT-PCR. **(P)** Bands of the protein expression levels of CTCF, DPP4, NLRP3, ASC, pro-caspase1, caspase-1-p20, pro-IL-1β, IL-1β, and GSDMD-N are detected by Western blot. **(Q)** Densitometry analysis of bands of the protein expression levels of CTCF, DPP4, NLRP3, ASC, pro-caspase1, caspase1-p20, pro-IL-1β, IL-1β, and GSDMD-N detected by Western blot. ns, non-significant, *p < 0.05, **p < 0.01, ***p < 0.001, and ****p < *0.0*001 in the comparison between the indicated groups.

To further validate the findings observed in the AML12 cell model of NAFLD, we established a NAFLD mouse model to test the hepatoprotective and lipid-lowering effects of gardenoside and CTCF silencing. The H&E staining results and the corresponding histological score showed that liver tissue in the normal control group had no obvious lesions; the liver lobules were intact, the cell cords were neatly arranged, and there was no infiltration of inflammatory cells around the blood vessels (Figures 9A,B). In the NAFLD model group, a large number of hepatocytes in the liver tissue were degenerated, the cytoplasm was filled with lipid droplets of different sizes, and inflammatory cell infiltration was seen in the liver lobules. The degeneration of liver tissue in the gardenoside and si-CTCF groups were reduced, and the degree of inflammatory cell infiltration around blood vessels was significantly improved (Figures 9A,B). Besides, the lipid-lowering effects of gardenoside and CTCF silencing were confirmed by Oil red O staining (Figure 9C). In addition, liver function indexes (ALT and AST) and blood lipid indexes (TG and TC) were also used to assess the liver function of mice in different treatment groups. The results revealed that compared with mice in the control group, these indexes were significantly increased in the NAFLD mice model, but the treatment of gardenoside or CTCF silencing reversed this trend (Figures 9D–G).

We also verified in the NAFLD mice model whether gardenoside inhibits NLRP3 inflammasome activation and hepatocyte pyroptosis through the CTCF/DPP4 signaling pathway. As shown in Figure 9H, gardenoside acted as a DPP4 inhibitor to reduce the activity of DPP4 in mice of the NAFLD model, and a similar observation was also made in the si-CTCF treatment group. In addition, gardenoside and si-CTCF treatment significantly reduced the content of intracellular ROS (Figure 9I) but also inhibited the content of downstream inflammatory factors IL-1β (Figure 9J) and IL-18 (Figure 9K). The RT-PCR analysis indicated that the mRNA levels of CTCF, DPP4, NLRP3 and ASC were increased in the NAFLD model mice but decreased by gardenoside and si-CTCF treatments (Figures 9L–O). Furthermore, the results of Western blot showed that the protein expression levels of CTCF, DPP4, ASC, NLRP3, caspase-1 p20, IL-1β, and GSDMD-N in the NAFLD model mice were upregulated compared with the mice in the control group, while in the mice treated with gardenoside or si-CTCF their expression levels were downregulated (Figures 9P,Q).

## Discussion

Gardenoside was previously proposed to be an inhibitor of NLRP3 inflammasome (Fu et al., 2020). A previous study illuminated that liraglutide could alleviate NLRP3-dependent pyroptosis through downregulating the expression of caspase-1 p20, IL-1β, and TNF-α, as well as attenuating the generation of ROS in H9c2 cells (Chen et al., 2018). Since NLRP3 inflammasome is considered a pivotal player in the progression of NAFLD (Wan et al., 2016), combined with the above evidence, it was hypothesized that gardenoside might regulate the pathogenesis of NAFLD *via* NLRP3 and its downstream targets. In addition, both DPP4 and CTCF have been proposed to participate in NAFLD, and we speculated that they might work in concert to induce multiple biological processes that promote the pathogenesis of NAFLD, and their synergy might be the target where gardenoside exerts its anti-NAFLD effects. Therefore, we first utilized two bioinformatics software to predict the binding site between DPP4 and CTCF. As a result, a highly matched binding site between CTCF and the promoter region of DPP4 was predicted, which was subsequently confirmed by dual-luciferase reporter assay system. The CTCF is an accepted transcription factor which exerts regulatory roles through binding to a specific target sequence (Ohlsson et al., 2001). Hence, we speculated that CTCF was also responsible for gardenoside mediated DPP4 suppression.

We next evaluated several NAFLD-associated markers in the AML12 cell model which underwent treatment with different concentrations of gardenoside in compliance with a previous report (Huang et al., 2017). It was then found that even 50 μM gardenoside could induce significant downregulation of CTCF and DPP4, which agreed with our previous hypothesis. We also analyzed the significance of other inhibited molecules (NLRP3, ASC, caspase1 p20, GSDMD-N, and IL-1β). NLRP3 has been proposed to be a target of several medications. For instance, liraglutide could ameliorate NASH through inhibition of NLRP3-induced pyroptosis (Yu et al., 2019). Saxagliptin intervention alleviated diabetic nephropathy by targeting NLRP3 in the rodent model (Birnbaum et al., 2016), and notably, gardenoside suppressed NLRP3 through autophagy in the microglial model (Fu et al., 2020). To the best of our knowledge, the current study provides the first evidence for the inhibitory effect of gardenoside on NLRP3 inflammasome in the context of NAFLD. The ASC is an adaptor protein that participates in the recruitment of pro-caspase-1 to NLRP3, which aggregates into an NLRP3-ASC-pro-caspase-1 complex and induces the cleavage of pro-IL-1β into its mature form (IL-1β). Thus, the abovementioned molecules were closely associated with NLRP3 inflammasome and jointly participated in pyroptosis (Yang et al., 2019). Another critical pyroptosis regulator was the N-terminal domain of GSDMD (GSDMD-N), which is a product of inflammatory caspase-1 induced cleavage (Shi et al., 2015). Taken together, downregulation of these markers indicated that NAFLD alleviated pyroptosis in NAFLD.

**Figure 10:**
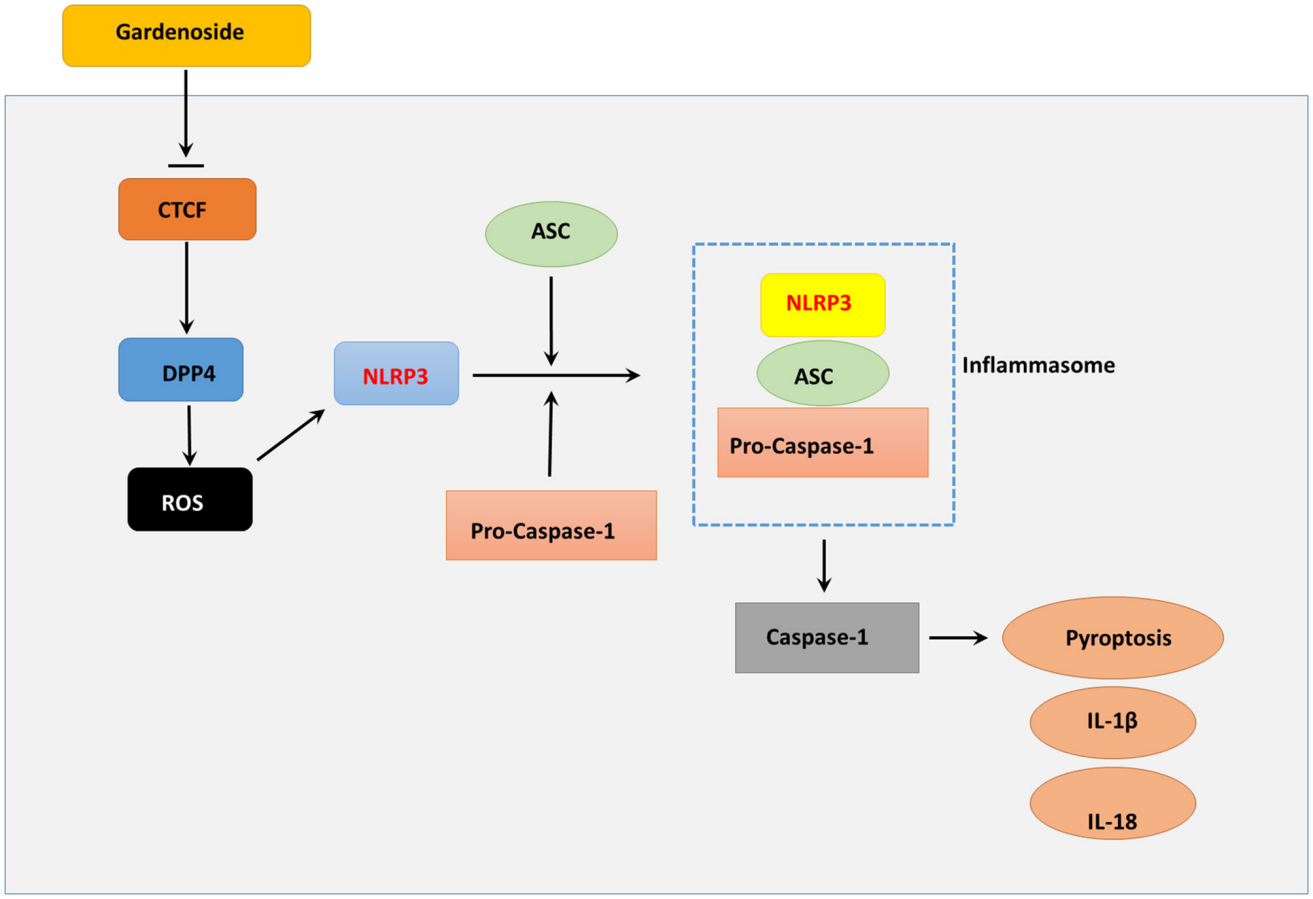
Summary diagram showing that gardenoside inhibits the activation of NLRP3 inflammasome and caspase-1-induced pyroptosis *via* CTCF/DPP4 signaling pathway.

Aside from cell viability which was important for reversing cellular impairments (Qiu et al., 2017) and pyroptosis that determined cell survival (Zheng and Li, 2020), excessive ROS production also played an important role in NLRP3 associated inflammation (Tschopp and Schroder, 2010). Excessive ROS production has been proposed to be regulated by DPP4 in endothelial cells (Ishibashi et al., 2013), and serves as a trigger for NLRP3 activation (Abais et al., 2015). For in-depth investigation, we evaluated the cell viability of NAFLD *in vitro* model using CCK8 assay and found that CTCF knockdown increased cell viability, mitigated pyroptosis, reduced ROS content in NAFLD *in vitro* model compared to control AML12 cells, which resembled the effects induced by 50 μM gardenoside treatment. These observations suggested that gardenoside could mitigate several NAFLD-induced impairments and, most importantly, CTCF knockdown and DPP4 silencing generated results similar to that induced by gardenoside. These altered biological characteristics coincided with several inhibited pro-pyroptotic molecules as described previously (DPP4, NLRP3, ASC, etc.). Therefore, it was hypothesized that gardenoside might improve NAFLD symptoms through targeting CTCF and several downstream targets of CTCF (Figure 10).

The liver serves as a fundamental organ for metabolizing lipid, where numerous regulators, including hormones and transcription factors, jointly participate in lipogenesis and subsequent lipid delivery and distribution (Nguyen et al., 2008). Perturbations in such a delicate regulating network might lead to lipid accumulation and culminate in the pathogenesis of NAFLD (Ipsen et al., 2018). In the current study, we evaluated lipid profile markers including levels of TC and TG and liver disease markers AST and ALT (Sookoian and Pirola, 2015) in serum collected from the NAFLD animal model and found a significant reduction of the above markers in the presence of either gardenoside, si-CTCF, or si-DPP4, accompanied by attenuated hepatic steatosis in an animal model and reduced lipid droplets accumulation in cell model as revealed by oil Red O and H&E staining, respectively. Consistently, gardenoside-, si-CTCF- and si-DPP4-induced improvements in the NAFLD animal model led to the inhibition of pro-pyroptotic molecules, which corroborated with the observations in the NAFLD cell model. The consistency across cell and animal models further substantiated that the CTCF-DPP4 cascade might be a target site for gardenoside. These observations were supported by protective roles of gardenoside through decreasing TG content of lipid droplets in another hepatic steatosis model, namely, free fatty acid-induced HepG2 cells (Liang et al., 2015).

Although significant results have been achieved in our current study, there are still some points that need further explanation, which might be addressed in our subsequent research: first, the current observations were based on laboratory models, clinical evidence regarding the effectiveness of gardenoside in the treatment of NAFLD and its mechanism of action *via* CTCF-DPP4 cascade is still lacking. In addition, the pharmacokinetic and pharmacodynamics of gardenoside are unclear. In-depth studies in these fields will further elucidate the potential of gardenoside as a candidate and CTCF-DPP4 cascade as a target for NAFLD therapy. In addition, *in vitro* study should include at least one more cell line other than mouse liver cell AML to support our conclusions. Another limitation is that though pathological changes were found in the NAFLD model, 12w HFD is not long enough to provoke NASH, this needs to be verified in future works.

## Conclusion

In this study, we first proposed a CTCF-DPP4 cascade identified by direct binding between CTCF and promoter region of DPP4, whereby CTCF and DPP4 synergistically promoted the pathogenesis of NAFLD through multiple pro-pyroptotic molecules, such regulatory network could be significantly inhibited by treatment with gardenoside. The therapeutic value of gardenoside was embodied by alleviated pyroptosis, attenuated hepatic steatosis, promoted cell viability, and reduced ROS production. These data may provide a novel avenue for NAFLD treatment and evidence for in-depth research on gardenoside.

## Data Availability Statement

The original contributions presented in the study are included in the article/supplementary material, further inquiries can be directed to the corresponding author/s.

## Ethics Statement

The animal study was reviewed and approved by Shanghai University of Traditional Chinese Medicine Animal Ethics and Use Committee.

## Author Contributions

TS made substantial contributions to drafting the manuscript, revising it critically for important intellectual content, and conception and design of the study. LC, B-BZ, and B-LX made substantial contributions to the acquisition, analysis, and interpretation of data. C-PZ and H-PW performed the experiments and analyzed data. All authors read and approved the version to be submitted.

## Funding

This work was supported by the Shanghai key medical specialty construction project (Grant Number ZK2019B16) and the independent innovation project of Shanghai Putuo District Health and Health Committee (Grant Number ptkwws201816). Clinical Specialized Discipline of Health System of Putuo District in Shanghai (grant number 2020TSZK01).

## Conflict of Interest

The authors declare that the research was conducted in the absence of any commercial or financial relationships that could be construed as a potential conflict of interest.

## Publisher's Note

All claims expressed in this article are solely those of the authors and do not necessarily represent those of their affiliated organizations, or those of the publisher, the editors and the reviewers. Any product that may be evaluated in this article, or claim that may be made by its manufacturer, is not guaranteed or endorsed by the publisher.
